# Induced Degradation of Tat by Nucleocapsid (NC) via the Proteasome Pathway and Its Effect on HIV Transcription

**DOI:** 10.3390/v5041143

**Published:** 2013-04-23

**Authors:** Hye-Won Hong, Seong-Wook Lee, Heejoon Myung

**Affiliations:** 1 Department of Bioscience and Biotechnology, Hankuk University of Foreign Studies, Yong-In, Gyung-Gi Do 449-791, Korea; E-Mail: hehe-wow@hanmail.net; 2 Department of Molecular Biology, Institute of Nanosensor and Biotechnology, Dankook University, Yong-In, Gyung-Gi Do 448-701, Korea; E-Mail: swl0208@dankook.ac.kr

**Keywords:** human immunodeficiency virus (HIV), nucleocapsid (NC), Tat, proteasomal degradation

## Abstract

Human Immunodeficiency Virus type 1 (HIV-1) is a retrovirus that causes acquired immunodeficiency syndrome (AIDS). HIV-1 Tat protein upregulates transcriptional transactivation. The nucleocapsid protein NC of HIV-1 is a component of virion and plays a key role in genome packaging. Herein, we have demonstrated the interaction between NC and Tat by means of a yeast two-hybrid assay, GST pull-down analysis, co-immunoprecipitation and subcellular colocalization analysis. We observed that the level of Tat was significantly reduced in the presence of NC. But NC did not affect mRNA expression level of Tat. The level of Tat in the presence of NC was increased by treating cells with a proteasome inhibitor, MG132. The ubiquitination state of Tat was not seen to increase in the presence of NC, suggesting the proteasomal degradation was independent of ubiquitination. Lowered level of Tat in the presence of NC led to a decrease in Tat-mediated transcriptional transactivation.

## 1. Introduction

It is estimated that there are 39 million AIDS patients worldwide. After 8–10 years of asymptomatic period, HIV causes devastation of the immune system leading to immunodeficiency. The virus infects immune cells including CD4^+^ T cells, dendritic cells and macrophages [1].

HIV Tat is a small nuclear protein encoded by an RNA consisting of two exons produced from multiple splicing [1]. It binds to the transcription activator response (TAR) element located at the 5' end of the viral RNA transcript and enhances transcription activity [2]. TAR RNA possesses a secondary structure consisting of a stem, bulge and loop, of which the bulge is the site where Tat binds [3]. It recruits human cyclinT1-Cdk 9 complex which is the component of a positive transcription elongation factor b (P-TEFb), to the loop [3]. Cdk 9 induces hyperphosphorylation of RNA polymerase II, resulting in enhanced transcription elongation. Tat also recruits p300/CBP and PCAF which are transcriptional coactivators/acetyltransferases to the promoter [4,5,6]. P-TEFb is then readily recruited to the promoter by acetylated Tat. 

Nucleocapsid (NC) is a 55 amino-acid basic protein produced from the processing of Gag precursor. It is a zinc finger protein containing two copies of CCHC motifs that is conserved among retroviruses [7]. This domain binds to the packaging signal (psi) at 5' LTR of the viral genomic RNA for encapsidation [8,9,10]. It possesses a nucleic acid chaperone activity which is crucial for the annealing of tRNA_3_^Lys^ and plus strand transfer [11]. It also plays a role in minus strand transfer by destabilization of TAR and the pausing of reverse transcription [11]. Another activity of NC is the induction of dimerization of viral genomic RNA in the virion core [12]. Virion NC was also shown to migrate from cytoplasm to nucleus and to control early viral mRNA [13]. Thus NC plays multiple roles in virus life cycle. 

The 55 KDa Gag precursor protein expressed from unspliced viral mRNA is transported to plasma membrane. After budding, Gag is cleaved by viral protease to produce p17 matrix, p24 capsid, p7 nucleocapsid, and p6 [14].

Herein is reported an additional role of NC based on the novel observation that NC and Tat interacted with each other. We hypothesize that NC from prematurely processed Gag may control transcriptional activation by Tat at a late stage of viral infection. 

## 2. Results and Discussion

**Figure 1:**
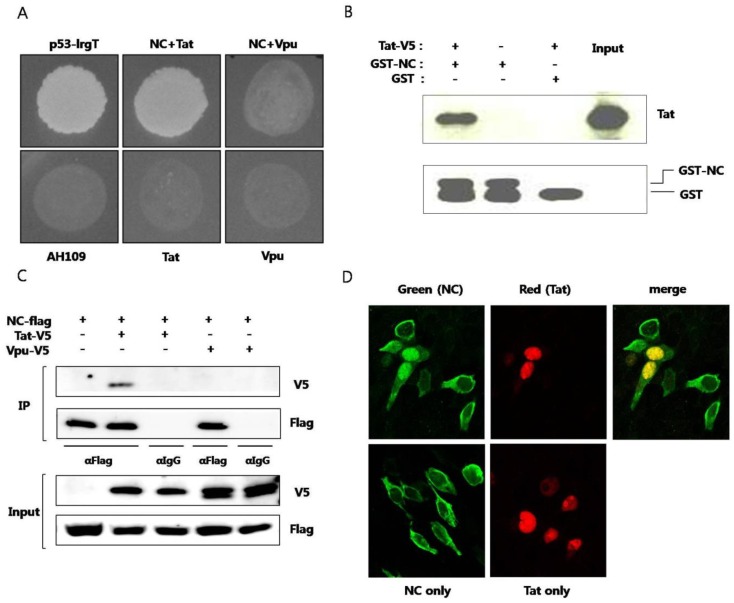
Interaction between HIV nucleocapsid (NC) protein and Tat (**A**) Plasmid expressing NC and plasmid expressing Tat were transformed to AH109 and the yeast was grown on media lacking histidine. Murine p53 and SV40 large T antigens were used as a positive control. HIV Vpu was used as a negative control. The lower panel shows yeast colonies resulting from interaction of the two proteins. Plasmids pGBK-NC, pGAD-Tat and pGAD-Vpu were constructed and utilized. pGBKT7-53 (murine p53) and pGADT7-T (SV40 large T antigen) were used as positive controls. Yeast strain AH109, transformed only with pGAD-Tat or pGAD-Vpu were used as negative controls; (**B**) Purified recombinant GST-fused NC was used to pulldown BKH21 cell lysate expressing Tat. GST was used as the negative control (third lane). pcDNA/V5-Tat was transfected to BHK-21 cells. After 24 h, a RIPA lysis buffer was added and lysis was performed at 4 °C for 30 min. The pellet was discarded after centrifugation and the lysate was obtained. GST or GST-NC and 30 μL of glutathione sepharose 4B bead was added to the lysate and the mixture was incubated at 4 °C overnight. HNGT buffer was used for washing and bound proteins were eluted after boiling with a 4XSDS sample buffer; (**C**) Flag-tagged NC and V5-tagged Tat or V5-tagged Vpu were expressed in HEK293 cells. The lysate was subjected to an immunoprecipitation with the anti-Flag antibody and detected with the anti-V5 antibody in a western blot analysis. The anti-IgG antibody was used a as the negative control; (**D**) Flag-tagged NC (green) and V5-tagged Tat (red) were expressed in HEK293 cells and observed under a confocal laser scanning microscope. At least three experiments were performed with essentially the same results.

Plasmid expressing NC and plasmid expressing Tat were cotransformed to yeast strain AH109 and the interaction of the two proteins were observed (Figure 1A). To confirm specific interaction between NC and Tat, HIV-1 Vpu protein was used as a negative control. Yeast cell cotransformed with NC and Vpu didn’t grow in SD media D.O. tryptophan, leucine, and histidine. Next, *in vitro* pulldown assay was performed. Purified recombinant GST-fused NC or GST alone was mixed with cell lysates of BHK-21 expressing Tat. The binding of Tat to GST-fused NC, not to GST, was observed (Figure 1B). GST pulldown of Tat was visualized by western blot. Coimmunoprecipitation was also used for observing the interaction between the two proteins. From the cell lysate expressing NC and Tat, the two proteins were immunoprecipitated together (Figure 1C). But, NC and Vpu proteins were not observed to interact with each other. Subcellular colocalization of the two proteins was also confirmed under laser scanning confocal microscope. It has been reported that virion NC localizes to the nucleus at the early infection stage [13]. In our experiments, NC was predominantly observed in the cytoplasm in the absence of Tat. It seemed that only part of overexpressed NC translocated to nucleus in the absence of Tat. However, in the presence of Tat, NC was shown to translocate to the nucleus and colocalize with Tat (Figure 1D). Both NC and Tat are translated in the cytoplasm and the binding between the two proteins seems to recruit NC to nucleus with their specific interaction. Tat has a nuclear localization signal (NLS) in the basic domain and mainly localizes in the nucleus, inducing transcription activation. The binding between the two proteins resulted in translocation of NC to the nucleus. 

**Figure 2:**
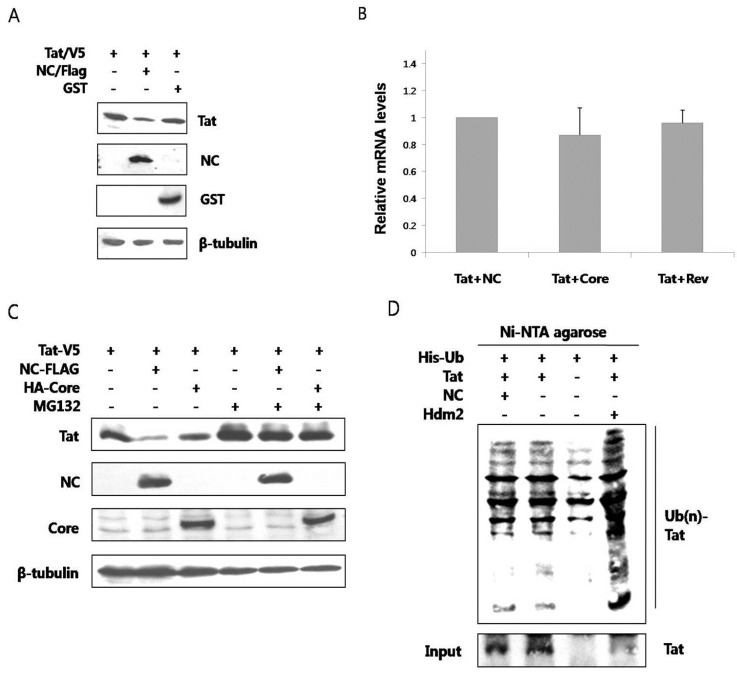
NC induced proteasomal degradation of Tat in an ubiquitin-independent pathway (**A**) V5-tagged Tat and Flag-tagged NC or GST were expressed in HEK293 cells and detected by a western blot analysis. Tat decreased in the presence of NC (middle lane), while GST had no effect; (**B**) Real time RT-PCR was performed to observe any change in the mRNA level of Tat in the presence or in the absence of NC. HCV core and HIV rev were used as the negative control; (**C**) V5-tagged Tat was expressed with Flag-tagged NC or HA-tagged HCV core in HEK293 cells. The decrease of Tat in the presence of NC was reversed when MG132, a proteasome inhibitor, was added to the culture (fifth lane); (**D**) HEK293 cells expressing His-tagged Ub, Flag-tagged NC, and HA-tagged Tat were grown in the presence of MG132 for 5 h at which time the lysate was passed through Ni-NTA agarose column. Western blot was performed with the anti-HA antibody. Hdm2 was used as a positive control. At least three experiments were performed with essentially the same results.

The outcome of interaction between the two proteins was explored. When the two proteins were co-expressed in HEK293 cells, the amount of Tat was observed to decrease through a western blot analysis (Figure 2A). Co-expression of GST did not alter the amount of Tat. To determine whether this change resulted from transcriptional control, we examined the amount of mRNA expressing Tat by reverse transcription PCR analysis. The amount of mRNA expressing Tat did not change in the presence or in the absence of NC (Figure 2B). Accordingly, Tat was hypothesized to degrade posttranslationally in the presence of NC. The primary degradation mechanism of proteins in eukaryotic cells involves the ubiquitin-proteasome pathway. Ubiquitin binds to unnecessary or misfolded proteins and this modification is recognized by the 26s proteasome leading to degradation of the target proteins [15]. To confirm whether the decrease of Tat in the presence of NC was via proteasomal degradation, MG132, a proteasome inhibitor, was used. The amount of Tat was seen to decrease less in the presence of NC when the cells were treated with MG132 (lanes 2 and 5, Figure 2C). As a comparison, the amount of Tat did not decrease in the presence of HCV core (lanes 3 and 6). The proteasome constitutively degrades nearly all proteins, so treatment with MG-132 is expected to increase the amount of Tat in the cell, appearing as if there was an increase in Tat expression. However, this is actually a decrease in Tat degradation. There is a decrease in Tat level with NC, but not alone or with HCV core protein, in untreated cells. This difference is alleviated when the proteasome is shut down, thus the proteasome is most likely causing the induced degradation of Tat by NC. To see whether ubiquitination occurred before the proteasomal degradation of Tat, ubiquitination assay was performed. In the presence of NC, the ubiquitination of Tat did not increase (Figure 2D). Hdm2, a proto-oncoprotein, is already known to induce the ubiquitination of Tat [16] and this was therefore used as the positive control in this experiment. 

**Figure 3:**
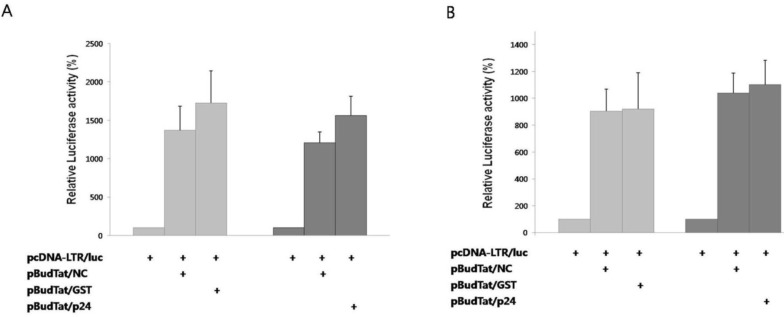
NC inhibited transactivation of Tat. (**A**) A luciferase reporter plasmid under the control of the HIV-1 LTR promoter was expressed with Tat in the presence of HIV NC, GST, or HIV p24; (**B**) Luciferase reporter activity was measured after treatment of cells with MG132. The mean value of three independent experiments is shown. Transcription efficiency was normalized with MUG assay.

Since Tat binds to the TAR RNA element and activates transcription elongation [2], we checked whether the degradation of Tat by NC affected this process. A reporter plasmid containing the luciferase gene under the control of HIV-1 LTR promoter was used. As negative controls, HIV p24, or GST was used instead of NC. In the presence of Tat, reporter activity increased notably (Figure 3A). In the presence of NC, reporter activity decreased 22% when compared to the tests in the presence of HIV p24 or GST. The decreased reporter activity by NC was effectively rescued by treatment with MG132 (Figure 3B). Thus, we have confirmed that the NC-mediated degradation of Tat results in the decrease of transcriptional activation.

There has been a report that HIV Tat is degraded by cellular p14^ARF^ in an ubiquitin-independent pathway [17]. In the latter study the degradation resulted in a decrease of transactivation. Many other investigations have reported that proteasomal degradation occurs independently of ubiquitination [18]. For example, tumor suppressor p53 is degraded by both the ubiquitin-dependent and ubiquitin-independent pathways [19]. There is also a report of viral transactivator degradation by its own viral protein. The hepatitis B virus transactivator X protein is degraded by both ubiquitin-dependent and ubiquitin-independent pathways [20]. Proteasomal degradation of HBV X protein induced by HBV core was suggested as a new mechanism of controlling virus life cycle [21].

Tat upregulates transcription from proviral genome. Transcribed early genes include Tat, Rev, and Nef. Late genes include Gag, Pol, Env, Vpr, Vpu, and Vif which are expressed in a Rev-dependent manner [22,23]. Although cleaved NC is generally found after proteolytic processing of Gag in released viral particles, premature processing of HIV Gag in cytoplasm is also reported [24]. A similar premature cytoplasmic processing of Gag was also shown in an avian retrovirus [25]. When NC is accumulated after premature processing in cytoplasm, it would have a chance to interact with Tat which was abundantly expressed at the early stage of infection. This interaction can lead to degradation of Tat via proteasomal pathway, leading to decrease in transcription at late stage of viral infection cycle. The 22% decrease of Tat-mediated transcription in the presence of NC in our experiment cannot be responsible for the entire silencing of transcription at the late stage. Since both NC and Tat were overexpressed in our experiment, Tat degradation effect would not be fully reflected. And there could be other mechanisms yet to be elucidated responsible for this transcriptional control. Nonetheless, the virus needs to minimize unnecessary transcription from the viral genome.

Based on our findings, we suggest an additional role of NC related to transcriptional control at the late stage of HIV replication. 

## 3. Experimental

### 3.1. Reporter Assay

For the transcription activity tests, BHK21 or HEK293 cells were used. Plasmids pcDNA/luc-LTR (0.5 μg), pBud-Tat/NC (2 μg), pBud-Tat/p24 (2 μg), pBud-Tat/GST (2 μg), and pCH110 (0.5 μg, Amersham Biosciences) were mixed with 8 μg of Lipofectamin 2000 (Invitrogen) and cells were incubated. After 19 h, MG132 (Calbiochem) was added at 10 μM to the mixture and incubated for 5 h. Luciferase assay mixture (Promega) was added and the activity was measured in a luminometer (Perkin Elmer). 

### 3.2. Proteasomal Degradation Assay

Cells were seeded in a 60 mm plate at 2 × 10^5^ cells and incubated for 18 h. Then 2 μg of pcDNA/V5-Tat, 0.5 μg of pCMV/Flag-NC and 0.5 μg of pcDNA-GST (or pcDNA-HA-core) were transfected to the cells using Lipofectamin 2000. After 18 h of incubation, 10 μM of MG132 (Calbiochem) was added to the mixture and incubated for a further 6 h.

### 3.3. Yeast Two Hybrid Assay

The Matchmaker two-hybrid system 3 (Clontech) was used. Plasmids pGBK-NC, pGAD-Tat and pGAD-Vpu were constructed and utilized according to the manufacturer’s protocol. pGBKT7-53 (murine p53) and pGADT7-T (SV40 large T antigen) were used as positive controls. Yeast strain AH109, trasformed only with pGAD-Tat or pGAD-Vpu were used as negative controls.

### 3.4. GST Pulldown

To obtain NC fused to GST, 1 liter of *E. coli* harboring pGNC1 was cultured and induced with 1 mM IPTG for 3 h. The protein was purified using affinity chromatography.

For GST pulldown analysis, 10 μg of pcDNA/V5-Tat was transfected to 5 × 10^5^ BHK-21 cells. After 24 h, a RIPA lysis buffer (50 mM Tris-Cl, pH 8.0, 1% NP-40, 0.5% sodium deoxycholate, and 150 mM NaCl) was added and lysis was performed at 4 °C for 30 min. The pellet was discarded after centrifugation and the lysate was obtained. Exactly 4 μg each of GST and GST-NC and 30 μL of glutathione sepharose 4B bead (Amersham Biosciences) were added to the lysate and the mixture was incubated at 4 °C overnight. Exactly 1 mL of HNGT buffer was used for washing and bound proteins were eluted after boiling with a 4×SDS sample buffer.

### 3.5. Coimmunoprecipitation

Exactly 8 μg of pcDNA/V5-Tat or pcDNA/V5-Vpu and 2 μg of pCMV/Flag-NC were used for transfection of HEK293 cells. Cells were lysed with a RIPA buffer in 24 h and anti-Flag antibody was added and further incubated at 4 °C overnight. Exactly 20 μL of protein G (Sigma-Aldrich) was added and the mixture was washed with lysis buffer 3 times. 

### 3.6. Subcellular Colocalization Analysis

Plasmids pcDNA/V5-Tat and pCMV/Flag-NC were used to transfect HEK293 cells. Cells were fixed and rabbit polyclonal anti-Flag antibody was added and incubated for 1 h. After washing 3 times, FITC-conjugated secondary antibody was added and the mixture was then incubated for 1 h. After washing 3 times, 1:200 diluted mouse monoclonal anti-V5 antibody was added. After 3 times of washing, TRITC-conjugated secondary antibody was incubated and the mixture was mounted on a glass plate and observed under a confocal scanning laser microscope (Zeiss).

### 3.7. Realtime RT PCR

Plasmids pcDNA/V5-Tat and pCMV/Flag-NC or pcDNA/HA-Core, pcDNA/HA-Rev were used for transfection of HEK293 cells. Cells were harvested after 24 h and total RNA was isolated using an RNA kit (Macherey-Nagel). Reverse transcription was performed using M-MLV reverse transcriptase (United States Biological) with the primer 5'-CTATTCCTTCGGGCCTGT-3'. Real-time PCR was performed with iQ SYBR Green supermix (Bio-Rad) for Tat and GAPDH gene expression. The real-time detection system was used to monitor the SYBR green signal at the end of each expression period for 40 cycles. Tat RNA levels were calculated by the delta/delta CT method with GAPDH genes. 

### 3.8. Ubiquitination Assay

Exactly 7 μg of pcDNA/HA-Tat, 2 μg of pCMV/Flag-NC and 5 μg of His-ubiquitin were used for the transient transfection of HEK293 cells. 40 hours after transfection, MG132 was added at 10 mM and the mixture was incubated for 5 h. Cells were then lysed with 1×RIPA buffer and subjected to sonication for 5 min. Supernatant was collected after centrifugation and loaded on Ni-NTA resin (Qiagen). After 5 h of binding reaction, the column was washed with 1×RIPA buffer 3 times and the eluate was analysed on an SDS-PAGE.

## 4. Conclusions

We showed the interaction between NC and Tat. The interaction induced nuclear translocation of NC and degradation of Tat via proteasomal pathway. Accumulation of prematurely processed NC in the cytoplasm can lead to decrease of Tat-mediated transcription.
